# Pathologies ORL et cervico-maxillo-faciales du sujet âgé en hospitalisation : aspects épidémiologiques, diagnostiques et thérapeutiques à Lomé, Togo

**DOI:** 10.48327/mtsi.v3i3.2023.336

**Published:** 2023-08-21

**Authors:** Winga FOMA, Palakina Pagninsi AGODA, Débora KOULETE, Essobiziou AMANA, Gérémie ANANIDJIN, Solim Uziel Roselin BOKO, Essobozou Plaodezina PEGBESSOU, Bathokédéou AMANA, Essohanam BOKO

**Affiliations:** 1 Service d'ORL et chirurgie cervico-maxillo-faciale du CHU Sylvanus Olympio de Lomé, Togo; 2 Service d'odontostomatologie du CHU Campus de Lomé, Togo; 3 Service d'ORL et chirurgie cervico-faciale du Centre hospitalier régional Lomé-commune, Togo; 4 Service d'ORL et chirurgie cervico-faciale du CHU Campus de Lomé, Togo

**Keywords:** Sujet âgé, Cellulite aiguë, Cancer du larynx, Gériatrie, Pathologies ORL, Hôpital, Lomé, Togo, Afrique subsaharienne, Elderly, Acute cellulitis, Laryngeal cancer, ENT pathologies, Geriatrics, Hospital, Lomé, Togo, Sub-Saharan Africa

## Abstract

**Introduction:**

Dans les pays en développement, 10% de la population est âgée de plus de 60 ans et cette proportion augmente rapidement. L'objectif g énéral de cette étude était de décrire le profil et la prise en charge du sujet âgé de 60 ans et plus, en hospitalisation au sein du service d'ORL et de chirurgie cervico-maxillo-faciale du CHU Sylvanus Olympio de Lomé, de 2012 à 2021.

**Méthode:**

Étude rétrospective descriptive. Après l'étude des dossiers, étaient sélectionnés les malades dont l'âge était de 60 années et plus, sans distinction de sexe, et hospitalisés pour une pathologie de la sphère ORL de janvier 2012 à décembre 2021. Étaient non inclus les patients âgés hospitalisés mais dont l'âge était imprécis, les dossiers vides ou très incomplets.

**Résultats:**

Au total, 296 dossiers ont été inclus représentant une fréquence hospitalière de 6,7%. L'âge moyen des patients était de 72 ans ± 7,3 ans avec des extrêmes de 60 ans et 95 ans. La tranche 60-69 ans représentait 59%. Le sex-ratio H/F était de 0,92. Les lésions de type infectieux/ inflammatoire étaient retrouvées chez 138 patients (46,6%) dont 38,9% étaient des cellulites aiguës d'origine dentaire, et les tumeurs étaient retrouvées chez 135 patients (45,6%). Les tumeurs étaient malignes chez 59 patients (44%) et bénignes chez 76 patients (56%). La thyroïde était le siège de 46 (61%) lésions bénignes et le larynx, le siège de 29 (49%) lésions malignes. La mauvaise hygiène bucco-dentaire, la prise d'anti-inflammatoires non stéroïdiens, le diabète et l'alcoolo-tabagisme étaient les facteurs favorisant la survenue des cellulites aiguës d'origine dentaire dans respectivement 89,6%, 80,9%, 20,0% et 2,6% des cas (p < 0,001). L'alcoolo-tabagisme était un facteur favorisant la survenue de tumeurs malignes dans 39% (p < 0,001). Un traitement chirurgical a été fait chez 234 patients (79,1%). Avec une létalité de 21%, la cellulite aiguë d'origine dentaire était la première cause de décès (p < 0,001).

**Conclusion:**

Les pathologies de la sphère ORL les plus fréquentes chez le sujet âgé dans notre contexte étaient infectieuses et tumorales, largement dominées par les cellulites aiguës d'origine dentaire, les adénomes thyroïdiens et le carcinome épidermoïde du larynx. Il s'avère important d'apporter de l'aide en matière de soins dentaires aux sujets âgés et de plaider en faveur du développement de la gériatrie au Togo.

## Abstract (en)

**Introduction:**

In developing countries, 10% of the population is over 60 years old and this proportion is increasing rapidly. The general objective of this study was to describe the profile and management of the elderly subject aged 60 and over, hospitalized in the ENT (Ear, Nose and Throat) and Head/Neck surgery department of the Sylvanus Olympio University Hospital in Lomé, from 2012 to 2021.

**Method:**

Retrospective descriptive study. After reviewing the files, selection of patients aged 60 and over, regardless of sex, and hospitalized for an ENT pathology from January 2012 to December 2021. Not included in our study were files of elderly subjects hospitalized but whose age was imprecise, or empty or very incomplete files.

**Results:**

A total of 296 cases were included, representing 6.7% of the department's activities. The mean age of the patients was 72 ± 7.3 years with extremes of 60 and 95 years. The age group 6069 years represented 59.5%. The sex ratio M/F was 0.92. Infectious/inflammatory lesions were found in 138 patients (46.6%) including 38.9% of acute cellulitis of dental origin, and tumors were found in 135 patients (45.6%). Tumors were malignant in 59 patients (43.7%) and benign in 76 patients (56.3%). Thyroid was the site of 46 (60.5%) benign lesions and larynx of 29 (49.2%) malignant lesions. Poor oral hygiene, use of non-steroidal anti-inflammatory drugs, diabetes and the combination of smoking and alcohol were the factors favoring the occurrence of acute cellulitis of dental origin in 89.6%, 80.9%, 20.0% and 2.6% of cases respectively (p<0.001). The combination of smoking and alcohol was a factor favoring the occurrence of malignancy in 39% (p<0.001). Surgical treatment was performed in 234 patients (79.1%). With a case lethality rate of 21%, acute cellulitis of dental origin was the leading cause of death (p<0.001).

**Conclusion:**

The most frequent pathologies of the ENT sphere in the elderly in our tropical context were infectious and tumoral, largely dominated by acute cellulitis of dental origin, thyroid adenoma and squamous cell carcinoma of the larynx. Despite the tendency to balance infectious and tumoral pathologies, the proportion of acute cellulitis of dental origin remains twice as high as that of malignant tumors. It contrasts with data from other sub-Saharan series where tumoral pathology remains predominant in the elderly. It is important to provide dental care assistance to the elderly and to advocate for the development of geriatrics and access to universal health coverage for the elderly in Togo.

## Introduction

La part de personnes âgées est en accroissement de par le monde [19]. Bien que définie de diverses manières selon les circonstances, la personne âgée a été définie par l'OMS comme une personne ayant dépassé l'espérance de vie médiane attendue à sa naissance [13]. Malgré cette variabilité dans la définition de cette frange de la population, l'âge de 60 ans et plus revient fréquemment dans les prévisions de l'OMS qui estime qu'entre 2015 et 2050, sa proportion va presque doubler au niveau mondial, passant de 12% à 22% [12]. En 2020, le nombre de personnes âgées de 60 ans et plus a dépassé celui des enfants de moins de 5 ans [12]. Dans les pays en développement, 10% de la population est actuellement âgée de 60 ans et plus, mais cette proportion augmente rapidement [2]. C'est en Afrique que le nombre de sujets âgés croît le plus rapidement, suivie par l'Amérique latine, les Caraïbes et l'Asie [21]. Le vieillissement s'accompagne en général de l'augmentation du nombre et de la gravité des affections et d'une dégradation de l'état de santé [10]. La pathologie oto-rhino-laryngologique (ORL) de cette population se singularise par son mode de présentation et par ses conséquences. Depuis quelques années, la littérature africaine a été enrichie par différentes études consacrées à la gériatrie ORL [14, 15, 16]. Celles-ci concourent à dresser le profil de la pathologie hospitalière ORL du sujet âgé et à analyser les facteurs liés à la mortalité dans cette frange de la population.

Une étude réalisée en 2013 dans un service d'ORL en Côte d'Ivoire avait estimé à 8% le taux d'hospitalisation des sujets âgés; leur âge moyen était de 67 ans et la pathologie prédominante était le carcinome épidermoïde du larynx (38,6%) [6].

Au Togo, en attendant le rapport du 5^e^ Recensement général de la population et de l'habitat (RGPH) qui s'est tenu du 24 octobre au 16 novembre 2022, celui du 4^e^ RGPH réalisé en 2010 avait estimé la proportion des personnes âgées de 60 ans et plus à 5,5% de la population togolaise et l'espérance de vie à la naissance à 60,8 ans [8]. Selon la Banque mondiale, l'espérance de vie au Togo est passée de 43 à 61 ans entre 1960 et 2020 [20]. Gbeasor-Komlanvi *et al.* dans leur étude évaluant la mortalité chez les personnes adultes âgées de 50 ans et plus au Togo dans les 3 premiers mois après leur admission à l'hôpital, avaient noté un haut taux de mortalité (17,2%) avec la majorité des décès qui sont survenus pendant l'hospitalisation [4]. Il n'existe pas de service de gériatrie au Togo. L'objectif général de cette étude était de décrire le profil et la prise en charge du sujet âgé de 60 ans et plus, en hospitalisation de 2012 à 2021 dans le service d'ORL et de chirurgie cervico-maxillo-faciale (ORL-CCMF) au CHU Sylvanus Olympio (CHU-SO) de Lomé, seul centre de référence de prise en charge en hospitalisation des pathologies ORL et CMF de la capitale togolaise.

## Méthode

### Type et période d'étude

Il s'est agi d'une étude observationnelle rétrospective portant sur les sujets âgés de 60 ans et plus, hospitalisés au sein du service d'ORL-CCMF du CHU-SO de Lomé. L'étude s'est déroulée de mars à juin 2022. Les données collectées étaient celles du 1^er^ janvier 2012 au 31 décembre 2021, soit une période de 10 ans.

### Matériel et population d'étude

Les données utilisées dans ce travail ont été recueillies à partir des dossiers des patients du service d'ORL-CCMF du CHU-SO de Lomé.

Après l'étude des dossiers, nous avons sélectionné les dossiers des malades dont l'âge était de 60 années et plus, sans distinction de sexe, et hospitalisés pour une pathologie de la sphère ORL.

Étaient non inclus dans notre étude les dossiers des sujets âgés hospitalisés mais dont l'âge était imprécis (le chiffre de l'âge varie dans les différentes parties du dossier), ou les dossiers vides ou très incomplets.

### Variables étudiées

Notre étude a pris en compte les variables suivantes:

- données sociodémographiques (âge, sexe, statut professionnel);
- antécédents pathologiques;
- siège et localisation des lésions;
- données étiologiques;
- durée d'hospitalisation;
- modalités du traitement ainsi que l'évolution.

### Définitions opérationnelles

Dans l'étude:

- les âges ont été groupés par tranches de 10 ans;
- le traitement médical a regroupé l'ensemble des médicaments reçus par le patient au cours de son hospitalisation, à l'exclusion des médicaments administrés au bloc opératoire.

### Collecte et analyse des données

La collecte des données a été faite à l'aide d'une fiche de recueil de données préétablie sur laquelle nous avons reporté les données épidémiologiques, diagnostiques et thérapeutiques concernant chaque malade. Nous nous sommes servis des registres d'hospitalisation pour recenser les patients âgés de 60 ans et plus, et ensuite des dossiers d'hospitalisation de ces patients préalablement recensés.

Le logiciel Epi Info version 7.2 a servi à la saisie et aux analyses statistiques des données. Le logiciel Excel 2016 nous a permis de faire les tableaux. La comparaison des fréquences des variables catégorielles a été faite à l'aide du test de Chi carré de Pearson et du test exact de Fischer. Les décisions ont été prises avec un seuil de risque de 5%.

### Considérations éthiques

L'autorisation N° 0599/2022/MSHPAUS/CHUSO/DIR/DRH/SERV.PERS a été obtenue des autorités sanitaires pour exploiter convenablement les dossiers des malades pour la présente étude. L'anonymat a été respecté pour chaque dossier.

## Résultats

### Caractéristiques sociodémographiques

Sur un total de 4 394 patients hospitalisés dans le service d'ORL-CCMF durant la période considérée, 306 étaient des personnes âgées de 60 ans et plus, dont 296 répondaient aux critères d'inclusion. Leur fréquence hospitalière était de 6,7%. L'âge moyen des patients était de 72 ans ± 7,3 ans avec des extrêmes de 60 ans et 95 ans. La tranche 60-69 ans représentait 59,5% (Tableau I).

**Tableau I:** Répartition des patients ≥ 60 ans selon les tranches d'âge, service ORL-CCMF, CHU Sylvanus Olympio de Lomé Distribution of patients ≥ 60 years old by age group, ENT and Head-Neck surgery department, Sylvanus Olympio Hospital of Lomé

	Effectif	Pourcentage
**[60 - 69[ans**	176	59,5
**[69 - 79[ans**	88	29,7
**[79 - 89[ans**	28	9,5
**≥ 89 ans**	4	1,4
**Total**	296	100

Il y avait 154 femmes (52%) soit un sexe-ratio H/F de 0,92. Les patients provenaient de la capitale Lomé dans 71,6% des cas (n = 212) et 84 venaient de l'extérieur de Lomé (28,4%). Deux cent dix-sept (217) patients (73,3%) étaient en activité dont 148 patients (68,2%) dans le secteur informel. Quarante-six (46) patients (15,5%) étaient des retraités de la fonction publique et 2 étaient encore en activité dans la fonction publique.

**Figure 1:**
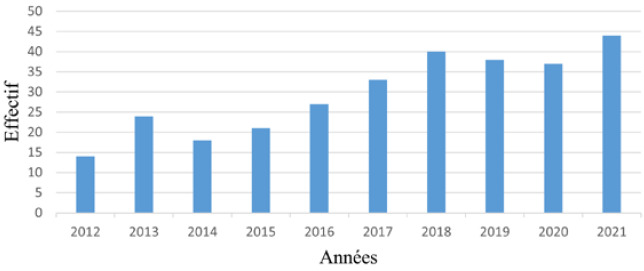
Répartition des patients âgés selon les années d'hospitalisation, service ORL-CCMF, CHU Sylvanus Olympio de Lomé) Distribution of old patients by years of hospitalization, ENT and Head-Neck surgery department, Sylvanus Olympio Hospital of Lomé

Nous avons observé une montée progressive de l'effectif d'hospitalisation au cours des années, avec un pic en 2021 avec 44 cas (14,9%) (Fig. 1). Le nombre d'hospitalisations mensuelles était de 24,6 avec des extrêmes de 14 en juillet et 30 en avril.

### Aspects diagnostiques

Les patients hospitalisés dans un contexte d'urgence étaient au nombre de 128 (43,2%) et ceux venus en consultation externe étaient au nombre de 168 (56,8%).

Les principaux motifs de consultation sont présentés dans le Tableau II.

**Tableau II:** Répartition des patients ≥ 60 ans selon les motifs de consultation, service ORL-CCMF, CHU Sylvanus Olympio de Lomé Distribution of patients ≥ 60 years old according to reasons for consultation, ENT department, Hospital Sylvanus Olympio of Lomé

	Effectif	Pourcentage
**Tuméfaction douloureuse**	129	43,6
**Odontalgie**	89	30,1
**Tuméfaction non douloureuse**	65	22,0
**Dyspnée laryngée**	47	15,9
**Dysphonie**	27	9,1
**Dysphagie**	22	7,4
**Rhinorrhée**	20	6,8
**Obstruction nasale**	15	5,1
**Céphalées**	15	5,1
**Odynophagie**	13	4,4
**Traumatisme facial**	12	4,1
**Gêne pharyngée**	3,0	1,0
Autres*	1,0	3,4

Dénominateur : 296

* Épistaxis, otalgie, acouphène, vertige, hypoacousie, anosmie

Une dénutrition a été observée chez 10 patients (3,4%) et 286 patients (96,6%) avaient un bon état nutritionnel. Un état général coté stade 0 selon la classification de l'OMS était retrouvé chez 174 patients (58,8%) (Tableau III).

**Tableau III:** Répartition des patients ≥ 60 ans selon l'état général, service ORL-CCMF, CHU Sylvanus Olympio de Lomé Distribution of patients ≥ 60 years old according to general condition, ENT and Head-Neck surgery department, Sylvanus Olympio Hospital of Lomé

	Effectif	Pourcentage
**Stade 0**	174	58,8
**Stade 1**	60	20,3
**Stade 2**	15	5,1
**Stade 3**	21	7,1
**Stade 4**	26	8,8
**Total**	296	100

Les lésions de type infectieux/inflammatoire étaient retrouvées chez 138 patients (46,6%) dont 38,9% étaient des cellulites aiguës d'origine dentaire, et les tumeurs étaient retrouvées chez 135 patients (45,6%). Une variabilité significative des types de lésions a été observée en fonction de l'âge (Tableau IV).

**Tableau IV:** Répartition des patients ≥ 60 ans selon le type de lésion et la tranche d'âge, service ORL-CCMF, CHU Sylvanus Olympio de Lomé Distribution of patients ≥ 60 years old by type of lesion and age group, ENT and Head-Neck surgery department, Sylvanus Olympio Hospital of Lomé

	[60-69[ans n (%)	[69-79[ans n (%)	[79-89[ans n (%)	≥ 89 ans n (%)	Total n (%)	p-value
**Infectieuse/inflammatoire**	80 (45,5)	40 (45,5)	16 (57,1)	2 (50)	138 (46,6)	
**Tumorale**	80 (45,5)	46 (52,3)	8 (28,6)	1 (25)	135 (45,6)	
**Traumatique**	15 (8,5)	1 (1,1)	2 (7,1)	1 (25)	19 (6,4)	
**Fonctionnelle et générale**	1 (0,6)	1 (1,1)	2 (7,1)	0	4 (1,4)	
**Total**	176	88	28	4	296	0,027

Cent vingt-trois patients (41,6%) avaient une lésion infectieuse/inflammatoire à la face (Tableau V).

**Tableau V:** Répartition des patients ≥ 60 ans selon le type de lésion et le siège, service ORL-CCMF, CHU Sylvanus Olympio de Lomé Distribution of patients ≥ 60 years old by type of lesion and location, ENT and Head-Neck surgery department, Sylvanus Olympio Hospital of Lomé

	Infectieuse / Inflammatoire	Tumorale	Traumatique	Fonctionnelle et générale
n (%)	n (%)	n (%)	n (%)
**Oreille**	5 (1,7)	1 (0,3)	0 (0)	0 (0)
**Fosses nasales**	3 (1,0)	6 (2,0)	0 (0)	2 (0,7)
**Sinus**	7 (2,4%)	5 (1,7)	0 (0)	0 (0)
**Face (parties molles et os faciaux)**	123 (41,6)	6 (2,0)	12 (4,0)	0 (0)
**Pharynx**	3 (1,0)	7 (2,4)	0 (0)	0 (0)
**Larynx**	2 (0,7)	35 (11,8)	0 (0)	2 (0,7)
**Téguments du cou**	30 (10,1)	8 (2,7)	0 (0)	0 (0)
**Cavité buccale**	53 (17,9)	11 (3,7)	0 (0)	0 (0)
**Thyroïde**	0 (0)	50 (17,9)	0 (0)	0 (0)
**Glandes salivaires**	3 (1,0)	4 (1,4)	0 (0)	0 (0)
**Œsophage**	0 (0)	2 (0,7)	07 (2,4)	0 (0)
**Thorax/thoraco-cervico-facial**	1 (0,34)	0 (0)	0 (0)	0 (0)

Dénominateur : 296

### Lésions infectieuses/inflammatoires

**Figure 2:**
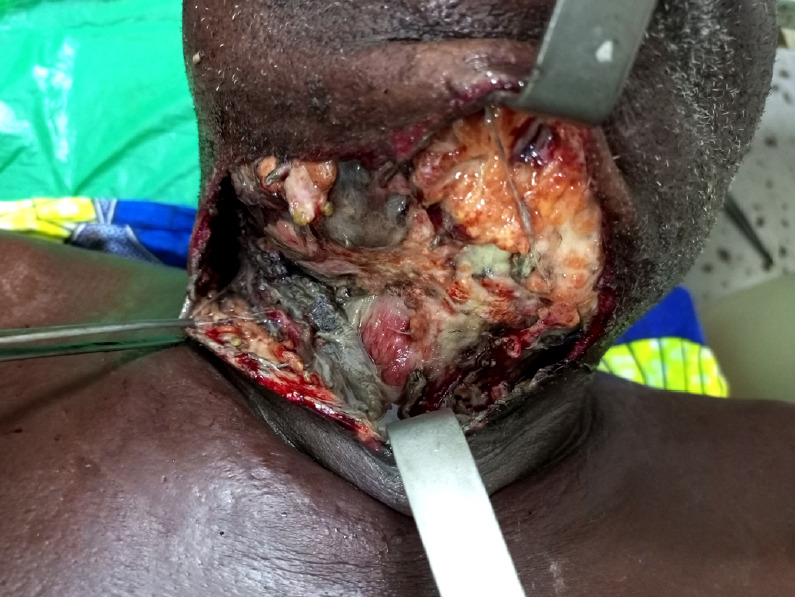
Cellulite cervicale nécrosante d'origine dentaire chez une personne âgée, service ORL-CCMF, CHU Sylvanus Olympio de Lomé Necrotizing cervical cellulitis of dental origin in an elderly person, ENT and Head-Neck surgery department, Sylvanus Olympio Hospital of Lomé

La cellulite aiguë d'origine dentaire (Fig. 2) venait en premier en ce qui concerne les lésions infectieuses chez 115 patients (83,3%). La carie dentaire était la cause de la cellulite aiguë dans 83 cas (72,2%); les autres cas de cellulite aiguë étaient survenus en post-extraction dentaire dans 32 cas (27,8%).

La mauvaise hygiène bucco-dentaire, la prise d'anti-inflaniniatoires non stéroïdiens, le diabète, le tabagisme et l'alcoolo-tabagisme étaient les facteurs favorisant la survenue des cellulites aiguës d'origine dentaire dans respectivement 89,6%, 80,9%, 20%, 5,2% et 2,6% des cas avec une relation statistiquement significative (Tableau VI).

**Tableau VI:** Répartition des cas de cellulite aiguë d'origine dentaire selon les facteurs favorisants chez des sujets ≥ 60 ans, service ORL-CCMF, CHU Sylvanus Olympio de Lomé Distribution of cases of acute cellulitis of dental origin according to contributing factors in patients ≥ 60 years old, ENT and Head-Neck surgery department, Sylvanus Olympio Hospital of Lomé

	Cellulite		p-value
	Oui	Non	
**Mauvaise hygiène bucco-dentaire**			
oui	103	19	< 0,001
non	12	162	
**Prise d'anti-inflammatoires non stéroïdiens**			
oui	93	19	< 0,001
non	22	162	
**Diabète**			
oui	23	9	< 0,001
non	92	172	
**Alcoolisme**			
oui	20	38	0,446
non	95	143	
**Tabagisme**			
oui	6	30	0,003
non	109	151	
**Alcoolo-tabagisme**			
oui	4	32	< 0,001
non	112	148	
**Virus de l'immunodéficience humaine**			
oui	2	1	0,320
non	113	180	

### Lésions tumorales

Les tumeurs étaient malignes chez 59 patients (43,7%) et bénignes chez 76 patients (56,3%).

**Figure 3:**
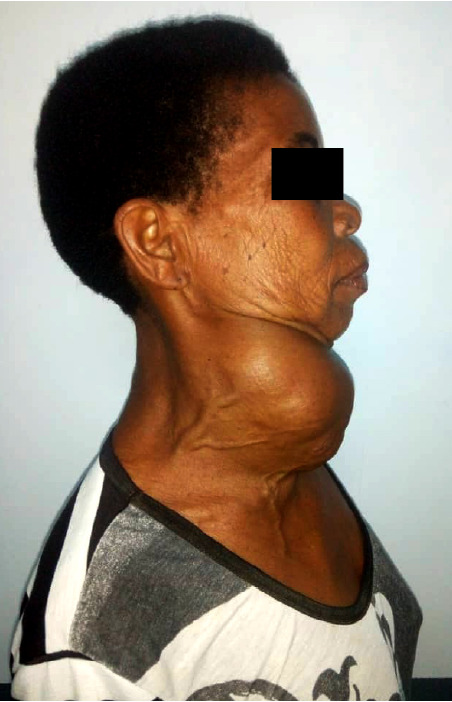
Volumineux goitre compressif chez une personne âgée, service ORL-CCMF, CHU Sylvanus Olympio de Lomé Voluminous compressive goiter in an elderly person, ENT and Head-Neck surgery department, Sylvanus Olympio Hospital of Lomé

La thyroïde était le siège de 46 (60,5%) des lésions bénignes (Fig. 3) et le larynx, le siège de 29 (49,2%) des lésions malignes.

L'alcoolo-tabagisme était un facteur favorisant la survenue de tumeurs malignes dans 39% des cas avec une relation statistiquement significative (Tableau VII). Le carcinome épidermoïde était retrouvé chez 51 patients (86%). Les adénomes étaient majoritaires parmi les tumeurs bénignes chez 49 patients (65%) (Tableau VIII).

**Tableau VII:** Répartition des tumeurs malignes en fonction de l'intoxication à l'alcool et au tabac chez des patients ≥ 60 ans, service ORL-CCMF, CHU Sylvanus Olympio de Lomé Distribution of malignant tumors according to alcohol and tobacco intoxication in patients ≥ 60 years old, ENT and Head-Neck surgery department, Sylvanus Olympio Hospital of Lomé

	Tumeurs malignes		p-value
	Oui	Non	
**Tabac seul**			
oui	08	28	0,738
non	51	209	
**Alcool seul**			
oui	10	48	0,882
non	49	189	
**Alcoolo-tabagisme**			
oui	23	12	< 0,001
non	36	225	

**Tableau VIII:** Répartition des patients selon le type histologique de la tumeur chez des patients ≥ 60 ans, service ORL-CCMF, CHU Sylvanus Olympio de Lomé Distribution of patients according to histological type of tumor in patients ≥ 60 years old, ENT and Head-Neck surgery department, Sylvanus Olympio Hospital of Lomé

	Effectif	Pourcentage
**Tumeurs malignes**	59	100
carcinome épidermoïde	51	86
carcinome papillaire	3	5
lymphome non hodgkinien	3	5
carcinome vésiculaire	1	2
carcinome basocellulaire	1	2
**Tumeurs bénignes**	76	100
adénome	49	65
papillome dysplasique	10	13
polype fibro-inflammatoire	7	9
hyperplasie folliculaire	3	4
améloblastome	3	4
angiome	2	3
ostéome	1	1
kyste sébacé	1	1

### Lésions fonctionnelles et générales

Nous avons observé quatre cas de lésions fonctionnelles réparties entre deux dysphonies dysfonctionnelles et deux épistaxis liées à une poussée d'HTA.

### Lésions traumatiques

Sur les 19 cas de lésions traumatiques, 12 cas étaient des fractures maxillo-faciales et 7, des corps étrangers pharyngo-œsophagiens. Les cas de corps étrangers étaient de type alimentaire chez 4 patients et inorganiques (prothèses dentaires) chez 3 patients.

### Aspects thérapeutiques

**Figure 4:**
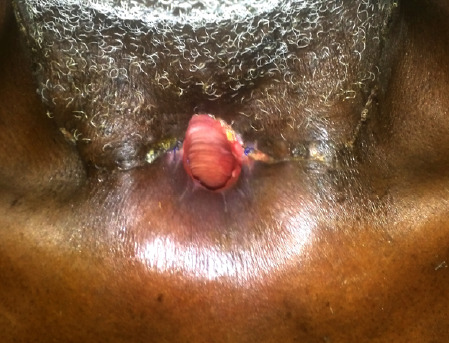
Tracheostome définitif après laryngectomie totale chez une personne âgée, service ORL-CCMF, CHU Sylvanus Olympio de Lomé Definitive tracheostoma after total laryngectomy in an elderly person, ENT and Head-Neck surgery department, Sylvanus Olympio Hospital of LoméLomé

Deux cent vingt patients (74,3%) étaient hospitalisés pour une durée comprise entre 0 et 10 jours. La médiane était de 9,3 jours avec des extrêmes de 1 et 60 jours. Un traitement médical était fait chez 292 patients (98,7%) et un traitement chirurgical chez 234 patients (79,1%). La radiothérapie, la chimiothérapie anticancéreuse et l'iode radioactif ont été faites chez respectivement 5, 2 et 2 patients. Une anesthésie générale a été faite chez 163 patients (59,7%) et une anesthésie locale chez 110 patients (40,3%). Une incision-drainage a été pratiquée chez 107 patients (45,7%) et une extraction dentaire chez 83 patients (35,5%). La laryngectomie totale (Fig. 4) a été pratiquée chez 21 patients (9%) (Tableau IX).

**Tableau IX:** Répartition des patients selon les gestes chirurgicaux chez des patients ≥ 60 ans, service ORL-CCMF, CHU Sylvanus Olympio de Lomé Distribution of patients according to surgical procedures in patients ≥ 60 years old, ENT and Head-Neck surgery department, Sylvanus Olympio Hospital of Lomé

	Effectif	Pourcentage
**Incision-drainage**	107	45,7
**Extraction dentaire**	83	35,5
**Thyroïdectomie**	50	21,4
**Laryngectomie + curage ganglionnaire**	21	9
**Intervention de Caldwell-Luc**	12	5,1
**Ostéosynthèse**	9	3,9
**Trachéotomie**	8	3,4
**Séquestrectomie**	6	2,6
**Amygdalectomie**	7	3
**Hémimandibulectomie**	3	1,3
**Submandibulectomie**	3	1,3
**Pharyngolaryngectomie + curage**	3	1,3
**Kystectomie**	1	0,4
**Maxillectomie**	1	0,4
**Polypectomie nasale**	1	0,4
**Parotidectomie totale**	1	0,4
**Ostéotomie**	1	0,4

Dénominateur : 234

### Aspects évolutifs

La guérison était observée chez 248 patients (83,8%) et le décès chez 38 patients, soit un taux de décès de 12,8%. Dix patients avaient signé une décharge pour une sortie d'hôpital contre avis médical (3,4%).

L'âge moyen des patients décédés était de 69,9 ans ± 7 ans avec des extrêmes de 60 ans et 85 ans. La durée moyenne d'hospitalisation des patients décédés était de 7,5 jours ± 10,3 jours avec des extrêmes de 1 et 60 jours.

La cellulite aiguë d'origine dentaire était la cause de décès chez 24 patients avec une relation statistiquement significative (p < 0,001). La mortalité des cellulites aiguës d'origine dentaire était donc de 8,1%, soit une létalité propre de 21%.

## Discussion

Cette étude a permis de décrire le profil et la prise en charge des pathologies ORL du sujet âgé de 60 ans et plus dans la capitale togolaise. Leur compréhension passe par la contextualisation du statut de la personne âgée. Comme pour beaucoup de pays pauvres, les Nations unies reconnaissent que la priorité va à l'avenir, c'est-à-dire aux enfants et aux jeunes et à la satisfaction de leurs besoins essentiels tels que la nourriture, la santé et l'éducation. Il est important de rappeler que le régime togolais de sécurité sociale est orienté vers les fonctionnaires et les travailleurs du secteur formel avec notamment les prestations familiales, les pensions d'invalidité ou de vieillesse, la prise en charge des accidents du travail et des maladies professionnelles. À côté de ces prestations, il faut noter d'autres sources de revenu soutenant les personnes âgées telles que les interventions ponctuelles des ONG, les transferts de fonds des enfants travaillant dans le pays ou dans la diaspora et le travail dans le secteur informel malgré l'âge. Il n'est pas rare que le sujet âgé soit lui-même la principale source de revenu de la famille, comme en témoigne la forte proportion de sujets encore en activité dans notre étude après 60 ans, âge d'admission à la retraite fixé par l'État togolais, avec quelques variations selon les secteurs professionnels. Le Togo envisage d'ailleurs de favoriser une insertion des personnes âgées à travers le volontariat et la relance du dialogue intergénérationnel conformément à la convention adoptée en 2016 sur la personne âgée lors de la 26^e^ session ordinaire de la conférence de l'Union africaine à Addis-Abeba en Éthiopie. Les difficultés matérielles des personnes âgées en milieu urbain sont l'un des facteurs intervenant dans les pathologies décrites.

Cette étude présente des limites qui tiennent au caractère rétrospectif de la collecte des données et à la situation géographique du centre de prise en charge constituant ainsi un biais dans le recrutement des patients. Avec plus de 70% des patients venant de la zone urbaine, il est clair que cette localisation occulte les cas pouvant provenir de régions éloignées. Avec une fréquence hospitalière à 6,7%, la proportion de personnes âgées de 60 ans et plus paraît faible dans notre série mais reste comparable à celle de la population générale au Togo qui était évaluée à 5,5% en 2010 [8]. Lawson-Ananissoh *et al.,* en étudiant les maladies digestives de la personne âgée en hospitalisation à Lomé, avaient rapporté une fréquence hospitalière de 17,8% [9]. La proportion de sujets âgés pourrait donc être sous-estimée. Selon la Banque mondiale, dans les pays en développement, la précarité dans laquelle vit une part des populations les empêche de venir en consultation [3]. Même en zone urbaine, cette précarité entraîne souvent des retards de consultation après des traitements traditionnels inadaptés aboutissant à des lésions localement avancées, comme en témoignent les Figures 2 et 3.

Dans la présente étude, nous avons observé une montée progressive de la courbe d'hospitalisation (Fig. 1) au cours des années. Ce résultat pourrait s'expliquer par le fait qu'en attendant la couverture sanitaire universelle, l'Institut national d'assurance maladie (INAM) œuvre depuis 2012 pour une meilleure couverture sanitaire des travailleurs des secteurs publics et de leurs familles. Ces dernières années, l'INAM a généralisé la couverture sanitaire aux travailleurs des secteurs formel et informel, ainsi qu'à leurs familles, favorisant ainsi l'accès aux soins [7].

Sur le plan diagnostique, malgré la tendance à l'équilibre entre les pathologies infectieuses et tumorales, la proportion de cellulite aiguë d'origine dentaire (38,9%) reste deux fois supérieure à celle des tumeurs malignes (19,9%) dans notre étude. Cette tendance caractérise de nombreux pays en développement en milieu tropical, avec une prédominance des infections graves mais évitables. A. J. Mercer [11] reconnaît que l'intervention humaine a entraîné une transition épidémiologique caractérisée par une baisse de la mortalité infantile, une augmentation de l'âge au décès, une augmentation de la durée de l'espérance de vie, une proportion plus élevée de personnes âgées dans la population et une réduction de la proportion de décès pour lesquels une maladie infectieuse est enregistrée comme cause sous-jacente. Toutefois des revers sont subis par des populations matériellement pauvres ou isolées qui font face à des maladies infectieuses graves. La gravité des cellulites aiguës d'origine dentaire est mise en évidence par leur létalité de 21%. Dans la littérature, la létalité due aux cellulites dans la population générale se situe autour de 10%. Notre étude montre une létalité élevée chez le sujet âgé, traduisant la précarité des soins bucco-dentaires avec ses corollaires d'automédication poussée et de traitements inadaptés. Les cellulites ont pratiquement disparu dans les pays développés grâce à un accès plus généralisé aux soins. De ce fait, il existe très peu de publications récentes sur le sujet dans la littérature internationale et le peu qui existe émane des pays en voie de développement [5]. L'amélioration de l'état de santé des personnes âgées sur le plan ORL doit prendre en compte la mauvaise hygiène bucco-dentaire, l'automédication par anti-inflammatoires non stéroïdiens, le diabète, le tabagisme et l'alcoolo-tabagisme qui ont été les facteurs favorisants significatifs de la cellulite aiguë d'origine dentaire dans la présente étude.

Les tumeurs malignes ont été dominées par le carcinome épidermoïde du larynx avec un effet favorisant de la consommation combinée d'alcool et de tabac. L'alcoolo-tabagisme a déjà été rapporté dans la survenue des cancers du larynx au Togo [1, 17]. L'émergence des cancers est généralement rapportée en milieu tropical en développement à l'augmentation de l'espérance de vie. Comme dans notre étude, la tranche de 60-69 ans est celle du cancer laryngé dans la littérature. La prise en charge de ces cancers est encore très limitée dans notre pratique. Les taux de radiothérapie et de chimiothérapie sont bas à côté de la chirurgie. Au Togo, il n'existe pas à ce jour de protocole national de prise en charge des cancers, encore moins de prise en charge du coût des traitements anticancéreux, les dépenses étant à la charge du patient ou de sa famille. Le seul centre de radiothérapie dans le pays est privé et n'est opérationnel que depuis 2 ans. La pathologie tumorale bénigne, 26% dans notre série, était essentiellement liée à la thyroïde. Le vieillissement est associé à des modifications complexes de la fonction thyroïdienne. Les carences nutritionnelles et les interactions médicamenteuses sont parfois à l'origine de pathologie thyroïdienne chez le sujet âgé [18].

La prise en charge hospitalière a nécessité dans la plupart des cas une hospitalisation de moins de 10 jours. La relative jeunesse de notre échantillon (près de 60% entre 60 et 69 ans) et l'état général conservé (près de 80% au stade 0 et 1 de l'OMS) pourraient avoir contribué à la bonne évolution chez la plupart des patients.

## Conclusion

Les pathologies les plus fréquentes étaient infectieuses et tumorales, largement dominées par les cellulites aiguës d'origine dentaire, les adénomes thyroïdiens et le carcinome épider-moïde du larynx. L'alcoolo-tabagisme est un facteur de risque dans la survenue de tumeurs malignes dans notre contexte. Le taux de décès reste relativement élevé, lié le plus souvent à la cellulite aiguë d'origine dentaire. Il s'avère important d'apporter de l'aide en matière de soins dentaires, de soutenir les campagnes de sensibilisation contre les infections bucco-dentaires et les cancers, de plaider en faveur du développement de la gériatrie et de l'accès à une couverture maladie universelle pour les personnes âgées au Togo.

## Contribution des auteurs

Conception de l'étude : Winga FOMA, Débora KOULETE.

Collecte et analyse des données : Débora

KOULETE, Essobiziou AMANA, Gérémie

ANANIDJIN, Solim Uziel Roselin BOKO.

Rédaction du manuscrit : Winga FOMA, Débora

KOULETE, Palakina Pagninsi AGODA.

Supervision, correction du manuscrit : Essobozou P. PEGBESSOU, Bathokédéou AMANA, Essohanam BOKO.

## Liens d'intérêt

Les auteurs ne déclarent aucun conflit d'intérêts.
